# Gastric Antral Vascular Ectasia as the First Presentation of Primary Biliary Cholangitis

**DOI:** 10.7759/cureus.21676

**Published:** 2022-01-27

**Authors:** Sugata N Biswas, Anshuman Elhence, Vinita Agrawal, Uday C Ghoshal

**Affiliations:** 1 Department of Gastroenterology, Sanjay Gandhi Post Graduate Institute of Medical Sciences, Lucknow, IND; 2 Department of Pathology, Sanjay Gandhi Post Graduate Institute of Medical Sciences, Lucknow, IND

**Keywords:** gastric antral vascular ectasia, argon plasma coagulation, portal hypertensive gastropathy, primary biliary cirrhosis, anti-mitochondrial antibody, portal hypertension

## Abstract

Primary biliary cholangitis (PBC), a chronic, autoimmune, cholestatic disease, typically occurs in elderly women and commonly presents with pruritus, fatigue, and cholestasis and its complications. Gastric antral vascular ectasia (GAVE), an uncommon cause of upper gastrointestinal bleeding, leading to transfusion-dependent chronic iron deficiency anemia, as the first presentation of PBC is unusual. We present the case of an elderly female with recurrent melena and transfusion-dependent anemia for a year without any history of jaundice, ascites, or hepatic encephalopathy. Investigations revealed iron-deficiency anemia, elevated transaminases, alkaline phosphatase (ALP), coarse liver, splenomegaly, and portal vein dilatation on ultrasound. An endoscopic evaluation revealed erythematous linear stripes in the antrum suggestive of GAVE, without esophageal or gastric varices. FibroScan (Echosens, Paris, France) revealed advanced F3 fibrosis. Further etiological workup showed positive antinuclear and antimitochondrial antibodies, elevated IgM levels, and negative viral markers (hepatitis B, C, A, and E). Clinically significant portal hypertension was revealed by the hepatic venous pressure gradient (HVPG), while transjugular liver biopsy (TJLB) revealed lymphocytic infiltration of bile duct epithelium with the destruction of small and medium-sized bile ductules. Iron supplementation, low-dose ursodeoxycholic acid, and argon plasma coagulation were used to treat the patient. At the three-month follow-up, no melena was reported and her hemoglobin and liver function tests remained normal. Patients with PBC presenting with GAVE and recurrent melena as a presenting symptom are rarely reported. An awareness of this presentation is important for its early diagnosis and effective treatment.

## Introduction

Primary biliary cholangitis (PBC) is a chronic, autoimmune, cholestatic disease characterized by progressive destruction of intrahepatic bile ducts. It mostly occurs in women in their fifth or sixth decades of life and frequently presents with pruritus, fatigue, and concurrent autoimmune disease(s) [1]. Gastric antral vascular ectasia (GAVE) is an uncommon but significant cause of upper gastrointestinal bleeding, often leading to transfusion-dependent chronic iron deficiency anemia that predominantly occurs in females. Though an association exists between PBC and GAVE, GAVE manifesting as recurrent melena as the first presentation of PBC is rarely reported in the literature [2].

## Case presentation

A 59-year-old female presented to the outpatient wing of the Department of Gastroenterology with a history of recurrent melena for a year. Before the presentation, she had been evaluated by a local physician and diagnosed with iron deficiency anemia. She had been diagnosed with hypertension 16 years ago and was on successful treatment with telmisartan 40 mg and chlorthalidone 6.25 mg daily. She denied having jaundice, ascites, or hepatic encephalopathy in the past. She further denied any history of surgery, tattooing, high-risk behavior, pruritus, alcohol consumption, and complementary or alternative medicine intake. There was no history of non-steroidal anti-inflammatory drug intake either. She denied any hematemesis and postural symptoms. She had not received any blood transfusion prior to the onset of melena.

**Figure 1:**
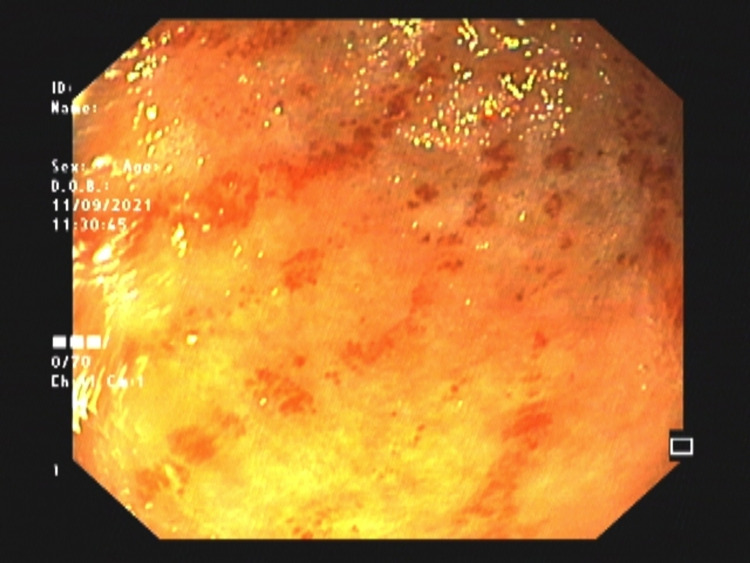
Upper gastrointestinal endoscopy showing reddish stripes in antrum suggestive of gastric antral vascular ectasia

At presentation, on general examination, she had pallor. Systemic examination did not reveal any abdominal organomegaly or ascites. Other system examinations were non-contributory. Laboratory examination revealed iron deficiency anemia [hemoglobin: 8.1 g/dL (normal range: 12-15 g/dL), serum iron: 12 g/dL (normal range: 60-170 g/dL), total iron-binding capacity: 502 g/dL (normal range: 240-450 g/dL), serum ferritin: 7 ng/mL (normal range: 12-260 ng/mL)]; she had a total leukocyte count of 5,100/mm^3^ (normal range: 4,000-11,000/mm^3^) with a differential of 61% neutrophils and 30% lymphocytes, and a platelet count of 189,000/mm^3^ (normal range: 150,000-400,000/mm^3^). Her liver function tests showed a total bilirubin level of 1.9 mg/dL (normal range: 0.3-1.2 mg/dL) with a direct fraction of 1.2 mg/dL, alanine aminotransferase (ALT) of 67 IU/L (normal level: <40 IU/L), aspartate aminotransferase (AST) of 221 U/L (normal level: <40 IU/L), alkaline phosphatase (ALP) of 261 U/L (normal level: <150 IU/L), total protein of 7.8 g/dL (normal range: 6.7-8.6 g/dL), and albumin level of 4.0 g/dL (normal range: 3.5-5.5 g/dL). Abdominal ultrasonography revealed a liver span of 12.6 cm (normal: 15.0 ± 1.5 cm in midclavicular line) with altered echotexture, mild splenomegaly, and a portal vein diameter of 13 mm (normal: <13 mm) without any evidence of ascites. She had required multiple blood transfusions over the last year, especially after each gastrointestinal (GI) bleeding episode. An upper GI endoscopy revealed GAVE without any evidence of esophageal or gastric varices. The patient had undergone three argon plasma coagulation (APC) sessions for GAVE before the current presentation. An upper endoscopy showed fiery-red linear stripes in the antrum suggestive of GAVE (Figure 1). Evaluation of small bowel as a possible source of GI bleeding was also undertaken with video capsule endoscopy and single balloon enteroscopy; however, both were non-contributory. FibroScan (Echosens, Paris, France) revealed a liver stiffness measurement (LSM) of 10.8 kPa (F3) (normal: <7 kPa) and a controlled attenuation parameter (CAP) of 215 dB/m (S1 steatosis: ≥263.5 dB/m).

**Figure 2:**
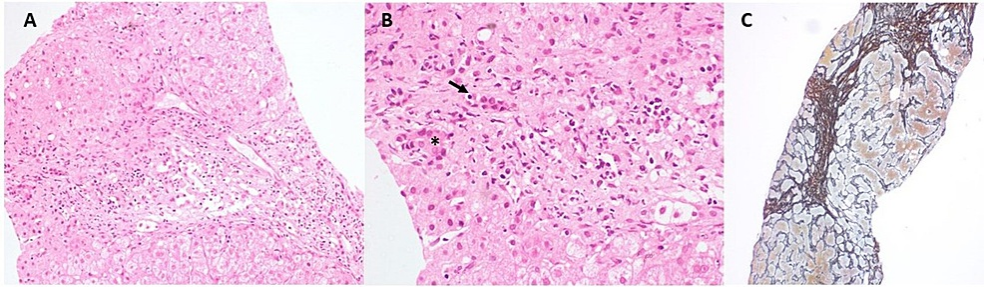
Liver histology of transjugular liver biopsy showing the expansion of portal tracts with the presence of a mixed inflammatory cell infiltrate and edema (A). There is infiltration of the bile duct epithelium by lymphocytes (arrow) and degenerative changes in the duct epithelium (asterisk) (B), consistent with a diagnosis of primary biliary cholangitis. Hepatic lobular architecture is distorted with the presence of porto-portal bridging fibrosis (C) Hematoxylin and eosin, A-200x, B-400x; reticulin, C-100x

In view of deranged liver function tests and advanced fibrosis (F3), further workup was undertaken to determine the underlying etiology of liver disease. Serological tests for hepatitis B, C, A, and E were negative. A battery of immunological tests for autoantibodies was remarkable for positive antinuclear antibody (ANA, 4+ at 1:100 titer) and antimitochondrial antibody (AMA, 4+ at 1:40 titer) in serum. Serum anti-smooth muscle antibody (ASMA) and anti-liver-kidney microsomal (LKM) antibodies were negative. Serum IgG at 1,750 mg/dL (normal range: 800-1,800 mg/dL) was normal while IgM level (624 mg/dL, normal range: 37-286 mg/dL) was raised. A transjugular liver biopsy (TJLB) and an assessment of hepatic venous pressure gradient (HVPG) were done. HVPG was 10 mmHg (free hepatic venous pressure: 18 mmHg, wedged hepatic venous pressure: 8 mmHg), which suggested clinically significant portal hypertension. TJLB showed distortion of the lobular architecture with the presence of porto-portal bridging fibrosis, infiltration of bile duct epithelium with lymphocytes along with degenerative changes and destruction of small and medium-sized bile ductules, expanded portal tracts with mixed inflammatory cell infiltrate comprising lymphocytes, plasma cells, eosinophils, and neutrophils without any evidence of cirrhosis, consistent with a diagnosis of PBC (Figures 2A-2C). With a diagnosis of PBC with portal hypertension and GAVE, the patient was treated with iron supplementation, APC, ursodeoxycholic acid, and beta-blocker. At three months of follow-up, she did not report having any further episodes of melena. Her liver function tests and hemoglobin levels returned to normal levels without further blood transfusion.

## Discussion

PBC is a chronic, progressive, autoimmune cholestatic disease characterized by inflammatory destruction of interlobar and septal bile ducts and positive AMA, a particular antibody considered as the serologic hallmark of the disease. Worldwide, the prevalence of PBC has seen an increase over the years, from 21.7 per 100,000 persons in 2004 to 39.2 per 100,000 persons in 2014, as reported by the Fibrotic Liver Disease Consortium Health Systems [3]. In India, PBC is extremely rare, with only a few case reports in the literature so far. The largest Indian series of 15 PBC patients showed that the most common presenting symptoms were pruritus (80%), jaundice (67%), skin changes (67%), and fatigue (60%) [4]. Interestingly, our patient did not complain of pruritus, which may indicate an Indian variant of PBC, as has been reported previously [5]. PBC is classically diagnosed in patients with cholestasis in the presence of raised ALP and positive AMA test and in the absence of extrahepatic biliary obstruction and other causes of liver disease. Chronic elevations in AST, ALT, ALP, total bilirubin with or without PBC-specific symptoms like pruritus and fatigue should also raise suspicion of PBC and necessitate further testing [6]. However, in cases where AMA is negative, cholestasis with specific ANA against antiglycoprotein (anti-gp) 210 and anti sp-100 may be diagnostic [7]. Liver biopsy is helpful in patients with diagnostic uncertainty or when the possibility of an additional superimposed entity like non-alcoholic steatohepatitis or autoimmune hepatitis confounds the diagnosis. Histological features like non-suppurative cholangitis and destruction of small or medium-sized bile ducts are highly suggestive of PBC [8]. PBC is a variably progressive disease with a risk of developing cirrhosis and its associated complications, including esophageal varices, ascites, and hepatic encephalopathy. However, it has been observed that portal hypertension may develop even in the absence of cirrhosis due to presinusoidal hypertension caused by nodular regenerative hyperplasia [9], as observed in our patient.

GAVE is an uncommon but significant cause of chronic GI blood loss [10]. Endoscopically, the lesions are characterized by either fiery red linear stripes radiating from the pylorus (watermelon stomach) or diffuse red spots involving the gastric mucosa (honeycomb stomach) [11,12]. Histologically, vascular ectasia of mucosal capillaries in association with fibrin thrombi, fibrohyalinosis, and spindle cell proliferation have been observed [13]. The occurrence of GAVE in association with PBC has been rarely reported in the literature. This association was first described in 1988 when two female patients with PBC who presented with chronic gastrointestinal blood loss and iron deficiency anemia were found to have GAVE on upper endoscopic evaluation [2]. In 1991, another report of a female patient with PBC with chronic transfusion-dependent anemia due to blood loss from GAVE was published, which was successfully managed by laser photocoagulation [14]. GAVE has also been described in a female patient of PBC who had concomitant limited systemic sclerosis [15]. In a study of 45 patients examining disease associations of GAVE, 20% of patients were found to have associated PBC. In contrast, 62% of patients had associated autoimmune or connective tissue disorders, including Raynaud’s phenomenon, sclerodactyly, sicca syndrome, hypothyroidism, and pernicious anemia [16]. However, as described in this report, GAVE with chronic transfusion-dependent iron deficiency anemia as the first presentation of PBC has been rarely reported [2], and requires a high index of suspicion on the part of the treating physician to clinch the diagnosis.

## Conclusions

PBC is characterized by progressive destruction of intrahepatic bile ducts and cholestasis, leading to disabling symptoms and complications of cirrhosis in middle-aged females. Timely institution of disease-modifying therapy slows the progression of the disease. Early diagnosis depends on identifying well-recognized symptoms of fatigue and pruritus and known associations with other autoimmune diseases. Although GAVE is known to be associated with PBC, it rarely appears as a presenting feature of the disease. Through this case report, we intend to educate physicians about this atypical presentation of PBC, which might aid in prompt and early diagnosis when encountered in clinical practice.

## References

[1] Younossi ZM, Bernstein D, Shiffman ML, Kwo P, Kim WR, Kowdley KV, Jacobson IM (2019). Diagnosis and management of primary biliary cholangitis. Am J Gastroenterol.

[2] Koivisto PV (1988). Gastric antral vascular ectasia and primary biliary cirrhosis. Endoscopy.

[3] Lu M, Zhou Y, Haller IV (2018). Increasing prevalence of primary biliary cholangitis and reduced mortality with treatment. Clin Gastroenterol Hepatol.

[4] Sarin SK, Monga R, Sandhu BS, Sharma BC, Sakhuja P, Malhotra V (2006). Primary biliary cirrhosis in India. Hepatobiliary Pancreat Dis Int.

[5] Sharma BC, Saraswat VA, Choudhuri G, Das A, Ghoshal UC, Pandey R (1996). Primary biliary cirrhosis without pruritus--an Indian variant. Trop Gastroenterol.

[6] European Association for the Study of the Liver (2017). EASL Clinical Practice Guidelines: the diagnosis and management of patients with primary biliary cholangitis. J Hepatol.

[7] de Liso F, Matinato C, Ronchi M, Maiavacca R (2017). The diagnostic accuracy of biomarkers for diagnosis of primary biliary cholangitis (PBC) in anti-mitochondrial antibody (AMA)-negative PBC patients: a review of literature. Clin Chem Lab Med.

[8] Lindor KD, Bowlus CL, Boyer J, Levy C, Mayo M (2019). Primary biliary cholangitis: 2018 practice guidance from the American Association for the Study of Liver Diseases. Hepatology.

[9] Colina F, Pinedo F, Solís JA, Moreno D, Nevado M (1992). Nodular regenerative hyperplasia of the liver in early histological stages of primary biliary cirrhosis. Gastroenterology.

[10] Dulai GS, Jensen DM, Kovacs TO, Gralnek IM, Jutabha R (2004). Endoscopic treatment outcomes in watermelon stomach patients with and without portal hypertension. Endoscopy.

[11] Papazian A, Braillon A, Dupas JL, Sevenet F, Capron JP (1986). Portal hypertensive gastric mucosa: an endoscopic study. Gut.

[12] Fuccio L, Mussetto A, Laterza L, Eusebi LH, Bazzoli F (2013). Diagnosis and management of gastric antral vascular ectasia. World J Gastrointest Endosc.

[13] Payen JL, Calès P, Voigt JJ (1995). Severe portal hypertensive gastropathy and antral vascular ectasia are distinct entities in patients with cirrhosis. Gastroenterology.

[14] Tsai HH, Smith J, Danesh BJ (1991). Successful control of bleeding from gastric antral vascular ectasia (watermelon stomach) by laser photocoagulation. Gut.

[15] Watson M, Hally RJ, McCue PA, Varga J, Jiménez SA (1996). Gastric antral vascular ectasia (watermelon stomach) in patients with systemic sclerosis. Arthritis Rheum.

[16] Gostout CJ, Viggiano TR, Ahlquist DA, Wang KK, Larson MV, Balm R (1992). The clinical and endoscopic spectrum of the watermelon stomach. J Clin Gastroenterol.

